# DeepMonitoring: a deep learning-based monitoring system for assessing the quality of cornea images captured by smartphones

**DOI:** 10.3389/fcell.2024.1447067

**Published:** 2024-08-27

**Authors:** Zhongwen Li, Lei Wang, Wei Qiang, Kuan Chen, Zhouqian Wang, Yi Zhang, He Xie, Shanjun Wu, Jiewei Jiang, Wei Chen

**Affiliations:** ^1^ Ningbo Key Laboratory of Medical Research on Blinding Eye Diseases, Ningbo Eye Institute, Ningbo Eye Hospital, Wenzhou Medical University, Ningbo, China; ^2^ National Clinical Research Center for Ocular Diseases, Eye Hospital, Wenzhou Medical University, Wenzhou, China; ^3^ Cangnan Hospital, Wenzhou Medical University, Wenzhou, China; ^4^ School of Electronic Engineering, Xi’an University of Posts and Telecommunications, Xi’an, China

**Keywords:** artificial intelligence, cornea image, deep learning, quality, smartphone

## Abstract

Smartphone-based artificial intelligence (AI) diagnostic systems could assist high-risk patients to self-screen for corneal diseases (e.g., keratitis) instead of detecting them in traditional face-to-face medical practices, enabling the patients to proactively identify their own corneal diseases at an early stage. However, AI diagnostic systems have significantly diminished performance in low-quality images which are unavoidable in real-world environments (especially common in patient-recorded images) due to various factors, hindering the implementation of these systems in clinical practice. Here, we construct a deep learning-based image quality monitoring system (DeepMonitoring) not only to discern low-quality cornea images created by smartphones but also to identify the underlying factors contributing to the generation of such low-quality images, which can guide operators to acquire high-quality images in a timely manner. This system performs well across validation, internal, and external testing sets, with AUCs ranging from 0.984 to 0.999. DeepMonitoring holds the potential to filter out low-quality cornea images produced by smartphones, facilitating the application of smartphone-based AI diagnostic systems in real-world clinical settings, especially in the context of self-screening for corneal diseases.

## Introduction

In recent years, the field of medicine has undergone a revolutionary transformation, driven by the advancements in artificial intelligence (AI), specifically deep learning (DL) which exhibits high accuracy in the automated classification of medical images, often at an expert level (Esteva et al., 2017; Hannun et al., 2019; Li et al., 2024; Rajpurkar et al., 2022). The application of DL in ophthalmology is very promising due to the fact that the diagnoses of eye diseases are mainly based on image recognition (Jiang et al., 2023; Li et al., 2022; 2023; Milea et al., 2020; Ting et al., 2019). Numerous studies have successfully developed DL systems for detecting retinal diseases from fundus images, such as diabetic retinopathy (DR), glaucoma, and retinal detachment (Dai et al., 2021; Li et al., 2021a; Li et al., 2020a; Pachade et al., 2021; Ting et al., 2017). Due to robust performance in clinical trials, the DL-based devices for DR screening and monitoring have been approved by the US and Chinese Food and Drug Administration and deployed in a large number of clinical institutions, particularly in primary care centers (Abramoff et al., 2018; Lin et al., 2021; Ting et al., 2020).

Recently, the prospect of using DL for corneal disease screening commands more attention because the report from the World Health Organization shows that blindness caused by corneal diseases is a major global ophthalmic public health concern (Flaxman et al., 2017). To increase the scalability of a corneal disease screening program while reducing its cost, several studies have constructed DL systems that can accurately detect corneal diseases from cornea images captured by smartphones (Li et al., 2021c; Wang et al., 2021; Yoo et al., 2021). This approach potentially brings corneal disease screening to high-risk patients rather than relying on patients to come to eye care institutions to be screened, enabling them to proactively identify their own corneal diseases at an early stage.

Despite exhibiting excellent performance during the research phase, the efficacy of smartphone-based AI systems in real-world settings remained uncertain as the studies initially excluded all low-quality images which was inevitable in real life and only high-quality images were used for model training and testing (Li et al., 2021b; Wang et al., 2021; Yoo et al., 2021). Several previous studies have confirmed that AI systems have greatly diminished performance in low-quality images (Li et al., 2020b; Li et al., 2021b; Maier et al., 2022). Patient-recorded images are often taken at various locations with different backgrounds and lighting, increasing the likelihood of insufficient clarity, exposure, or overexposure. It poses a challenge to those smartphone-based AI systems. Therefore, the adoption of an approach capable of minimizing or even preventing the occurrence of low-quality images is crucial to facilitate the implementation of smartphone-based AI systems, especially in the scenario of corneal disease self-screening. To date, the solution to this problem has not been investigated in the published research.

In the current study, we endeavored to tackle this issue by developing a DL-based image quality monitoring system, named DeepMonitoring, to automatically detect low-quality cornea images produced by smartphones. Meanwhile, this system could identify the cause of low image quality, assisting an operator in adopting targeted measures to obtain high-quality cornea images in a timely manner. Besides, to verify the generalizability of our system, it was tested on two independent datasets that were collected using different brands of smartphones.

## Methods

### Datasets

The development of DeepMonitoring involved the use of 6,374 cornea images captured with smartphones, formatted in JPG with dimensions of 4,680 × 3,456 pixels. These images were taken utilizing the super macro mode feature of the HUAWEI P30 camera at Zhejiang Eye Hospital (ZEH). Two additional datasets, including 1,343 smartphone-based cornea images obtained at Ningbo Eye Hospital (NEH), were used to externally assess DeepMonitoring. One dataset, consisting of 769 images (4,032 × 3,024 pixels in JPG format), was acquired using the super macro mode of VIVO X80. The second dataset, containing 574 images (2,592 × 1,944 pixels in JPG format), was collected using the super macro mode of XIAOMI 12S. All smartphone-based cornea images were de-identified before being transferred to researchers.

### Ethics approval

Ethical approval for this study was obtained from the Ethics Committees of Ningbo Eye Hospital (NEH) (identifier: 2022-56 K-C1) and Zhejiang Eye Hospital (ZEH) (identifier: 2021-019-K-16-03). The research adhered to the principles outlined in the Declaration of Helsinki. Due to the retrospective nature of data collection and the utilization of de-identified images, the requirement for informed consent was waived.

### Image quality classification and ground truth

**FIGURE 1:**
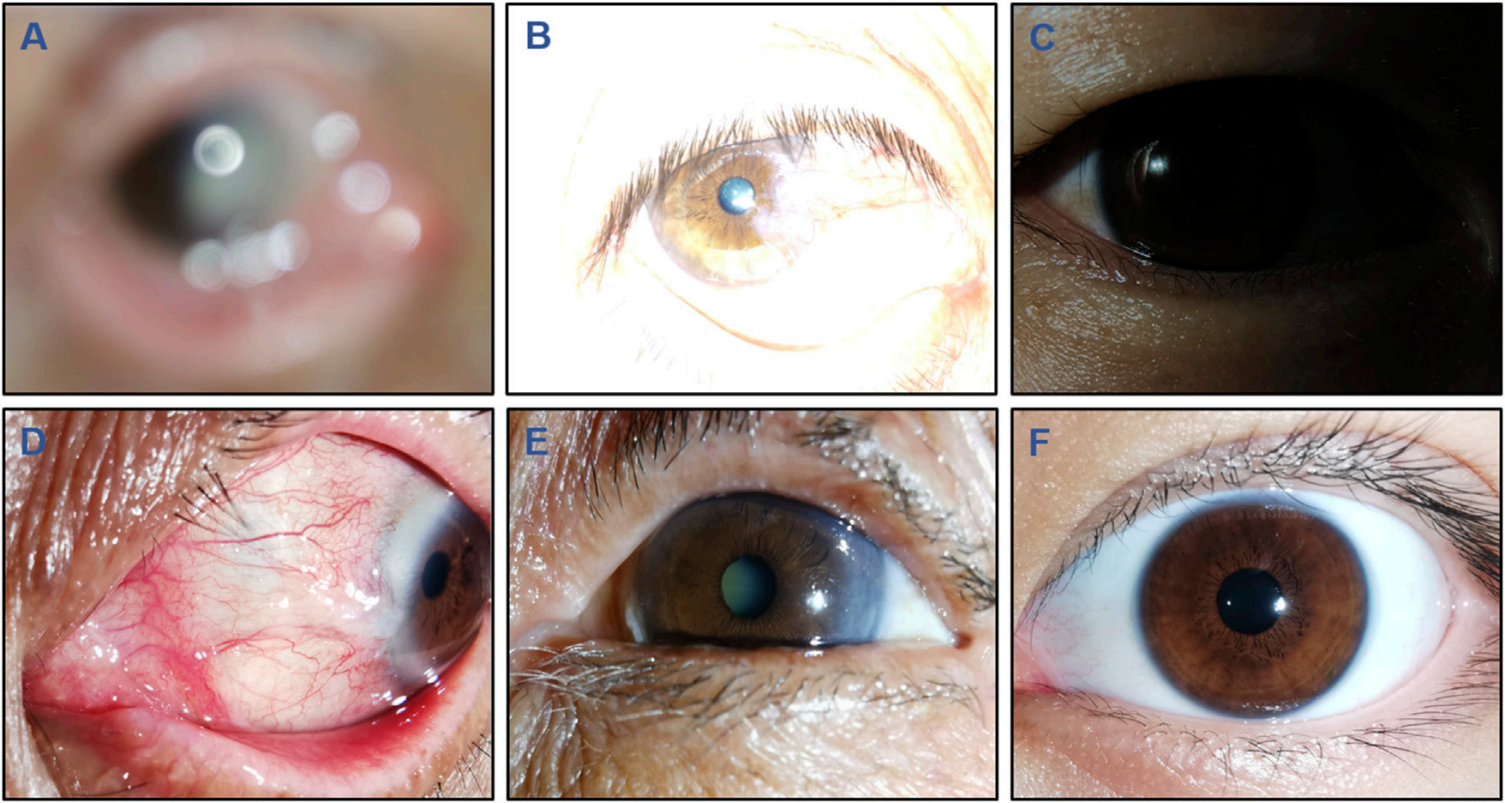
Typical examples of low-quality and high-quality cornea images taken by smartphones. Low-quality images include the following 5 types: **(A)**, Defocused image; **(B)**, Overexposed image; **(C)**, Underexposed image; **(D)**, Image of poor cornea position; and **(E)**, Image of incompletely exposed cornea. **(F)**, High-quality image.

The details of the criteria for image quality are shown in Table 1. Three cornea specialists, each possessing over 5 years of clinical experience, were engaged to independently annotate images according to the specified criteria. All images were classified into the following 6 categories: defocused images, overexposed images, underexposed images, images of poor cornea position (PCP), images of incompletely exposed cornea (IEC), and high-quality images. Typical examples of images with varying quality are presented in Figure 1. The ground truth of each image was established upon consensus among the 3 cornea specialists. Discrepancies at any level were resolved through the judgment of a senior cornea specialist with 20 years of clinical experience. The performance of DeepMonitoring (a multiclassification classifier) was evaluated on the basis of the ground truth.

**TABLE 1:** The classification of the quality of smartphone-based cornea images.

Classification	Presence of features
High-quality images	Four-fifths or more of the cornea is clear
Low-quality images	Meet any of the following criteria
Defocused images	Focus is not on the cornea
Overexposed images	Over one-fifth of the cornea is unclear due to overexposure
Underexposed images	Over one-fifth of the cornea is unclear due to underexposure
Images of poor cornea position	Over one-fifth of the cornea is indistinct because the cornea position is not straight ahead
Images of incompletely exposed cornea	Over one-fifth of the cornea is covered by eyelids

### Development and evaluation of DeepMonitoring

Before employing DL, image standardization was undertaken to resize the images to a resolution of 224 × 224 pixels and normalize pixel values within the range of 0–1. To enhance the diversity of the training set and mitigate overfitting and bias during the training process, data augmentation techniques were employed. All images in the training set underwent random cropping, rotations, as well as horizontal and vertical flipping, effectively increasing its size to six times the original.

Images from the ZEH dataset were randomly allocated 70% to the training set, 15% to the validation set, and the remaining 15% to the internal testing set for the development and performance evaluation of DeepMonitoring. To obtain the most optimal model with robust performance, the study explored four state-of-the-art DL architectures: Swin-Transformer, ConvNeXt, RepVGG, and MobileNet. The details of the trained DL models, including the size, trainable parameters, and running time of training and testing, are shown in Supplementary Table 1. Transfer learning was employed due to its potential to enhance the performance of DL models in image classification tasks (Kermany et al., 2018). The DL architectures were initialized using weights pre-trained on the ImageNet database, which consists of 1,000 object classes (Russakovsky et al., 2015).

The models were trained using the PyTorch backend based on an Ubuntu 18.04 computer equipped with four Nvidia 2080TI graphics processing units. The model underwent optimization utilizing the Adaptive Moment Estimation (ADAM) optimizer, with an initial learning rate set to 0.001 and a weight decay set to 5e-05. Throughout the training process, the monitoring of validation accuracy and loss was implemented as a precautionary measure to avoid model overfitting. Following each training epoch, the validation set was utilized to assess what the model had learned. Whenever the validation accuracy improved or the loss function decreased, a checkpoint was generated to preserve both the model architecture and its corresponding weight matrix. The maximum iteration was established at 80 steps, with a batch size set to 64. The model with the highest validation accuracy and the lowest validation loss was selected for application on the testing set.

**FIGURE 2:**
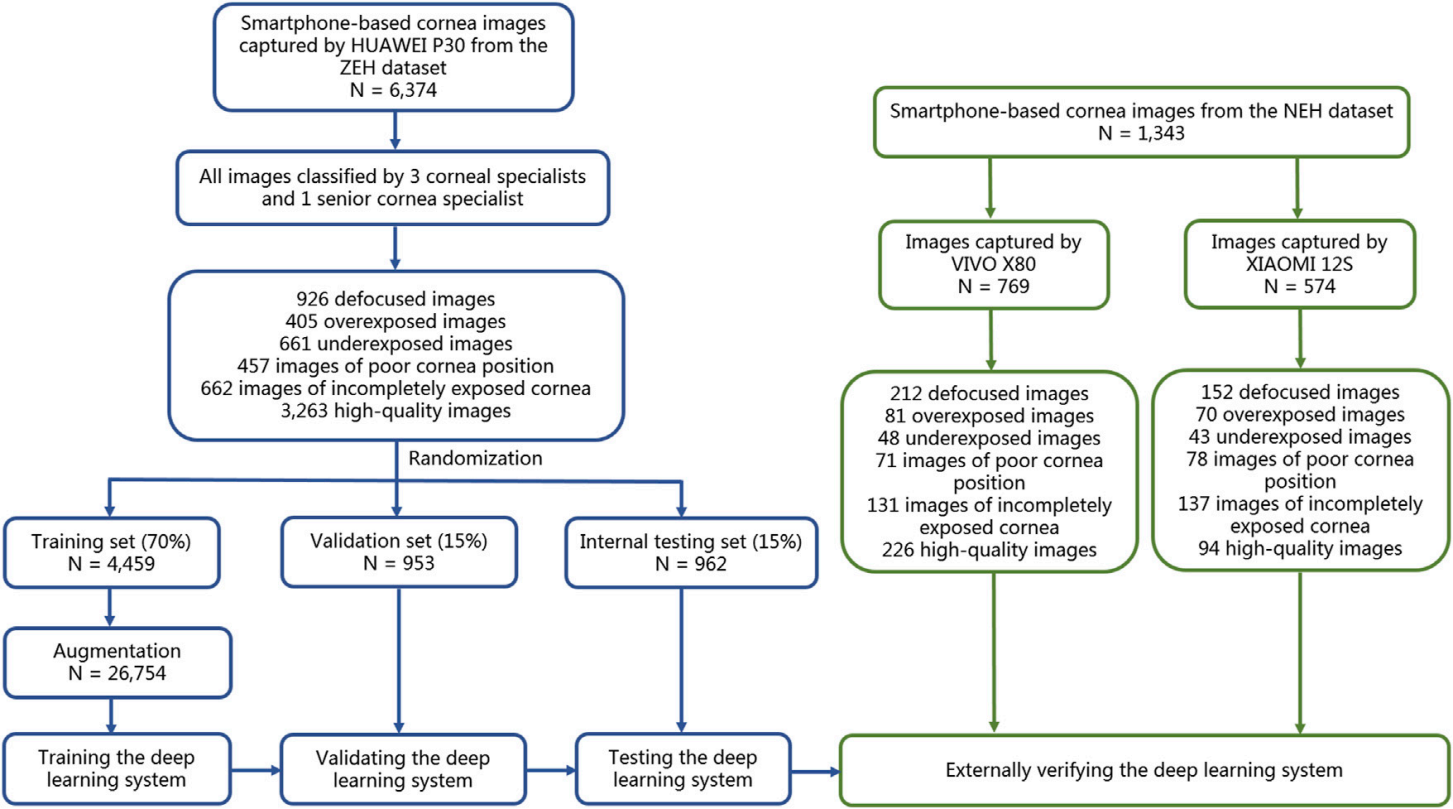
Flow diagram of the development and evaluation of the deep learning system. NEH, Ningbo Eye Hospital. ZEH, Zhejiang Eye Hospital.

The effectiveness of this six-category classification model was subsequently appraised on two external testing sets. The process of establishing and assessing DeepMonitoring is introduced in Figure 2. The t-distributed stochastic neighbor embedding (t-SNE) was employed as a nonlinear dimensionality reduction technique to showcase the embedding features learned by the DL model for each category in a two-dimensional space.

### Heatmap generation

To gain the trust of human experts in clinical practice, DeepMonitoring requires an understandable decision-making process. The Gradient-weighted Class Activation Mapping (Grad-CAM) technique (Selvaraju et al., 2020), as a generalization to CAM, was used to highlight the particular image regions that were most responsible for predicting a certain class by DeepMonitoring. Grad-CAM can be applied to a broader spectrum of DL model families without necessitating architectural changes or retraining. It generates heatmaps from the final convolutional layer in test images, assisting human experts in comprehending the rationale behind the DL system. The redder regions in the heatmaps indicate a higher influence on DeepMonitoring’s classification.

### Evaluation of an AI diagnostic system in low-quality and high-quality cornea images

We previously developed an AI diagnostic system for discriminating among keratitis, other corneal abnormalities, and normal cornea utilizing cornea images, which aimed to automatically detect corneal diseases at an early stage and decline the incidence of corneal blindness (Li et al., 2021b). Typical examples of keratitis images are shown in Supplementary Figure 1. To demonstrate the importance of the image quality assessment process (DeepMonitoring) for this AI diagnostic system, we conducted separate evaluations of the system’s performance using low-quality and high-quality cornea images. A set of 300 high-quality images was randomly sampled from the ZEH dataset, with each category (keratitis, other corneal abnormalities, and normal cornea) comprising 100 images. Using the same strategy, we selected 300 low-quality images and each category consisted of 20 defocused images, 20 overexposed images, 20 underexposed images, 20 PCP images, and 20 IEC images. The performance of the AI diagnostic system in low-quality images was contrasted with its performance in high-quality images. To determine which type of low-quality images (defocused images, overexposed images, underexposed images, PCP images, and IEC images) had the biggest negative impact on the AI diagnostic system, we evaluated this system using the low-quality images randomly selected above.

### Statistical analyses

DeepMonitoring’s performance in discriminating among defocused images, overexposed images, underexposed images, PCP images, IEC images, and high-quality images was assessed using the one-versus-rest strategy. The evaluation involved calculating accuracy, sensitivity, and specificity, along with 95% confidence intervals (CIs). Receiver operating characteristic (ROC) curves were generated to illustrate DeepMonitoring’s capability in evaluating image quality. The greater area under the curve (AUC) signified superior performance. Unweighted Cohen’s kappa coefficients were applied to assess the concordance between DeepMonitoring’s outcomes and the ground truth established by cornea specialists. The interpretation of Kappa agreement scores followed a predefined scale from a previously published source (0–0.20: none; 0.21–0.39: minimal; 0.40–0.59: weak; 0.60–0.79: moderate; 0.80–0.90: strong; > 0.90: almost perfect) (McHugh, 2012). Proportion comparisons were conducted utilizing the McNemar test. All statistical analyses were two-sided and performed using Python version 3.7.8 (Wilmington, Delaware, United States), with a significance level set at 0.05.

## Results

### Characteristics of the datasets

A total of 7,717 smartphone-based cornea images (1,290 defocused images, 556 overexposed images, 752 underexposed images, 606 PCP images, 930 IEC images, and 3,583 high-quality images) were utilized to develop and evaluate DeepMonitoring. Comprehensive details about the development and external testing datasets are presented in Table 2.

**TABLE 2:** Summary of datasets.

Item		Development dataset		External testing dataset	
Total no. of images		6,374		1,343	
Institution		ZEH		NEH	
Location of institution		Wenzhou		Ningbo	
Smartphone model		HUAWEI P30		VIVO X80	XIAOMI 12S
Image format		JPG		JPG	JPG
Image size (pixels)		4,680 × 3,456		4,032 × 3,024	2,592 × 1944
	Training set	Validation set	Internal testing set	VIVO external testing set	XIAOMI external testing set
Defocused images^a^	648/4,459 (14.5)	138/953 (14.5)	140/962 (14.6)	212/769 (27.6)	152/574 (26.5)
Overexposed images^a^	283/4,459 (6.3)	60/953 (6.3)	62/962 (6.4)	81/769 (10.5)	70/574 (12.2)
Underexposed images^a^	462/4,459 (10.4)	99/953 (10.4)	100/962 (10.4)	48/769 (6.3)	43/574 (7.5)
Images of poor cornea position^a^	319/4,459 (7.2)	68/953 (7.1)	70/962 (7.3)	71/769 (9.2)	78/574 (13.6)
Images of incompletely exposed cornea^a^	463/4,459 (10.4)	99/953 (10.4)	100/962 (10.4)	131/769 (17.0)	137/574 (23.8)
High-quality images^a^	2,284/4,459 (51.2)	489/953 (51.3)	490/962 (50.9)	226/769 (29.4)	94/574 (16.4)

^a^ Data are no. of images/total no. (%) unless otherwise indicated. ZEH, Zhejiang Eye Hospital; NEH, Ningbo Eye Hospital; JPG, joint picture group.

### Performance of DL models in a validation set

**FIGURE 3:**
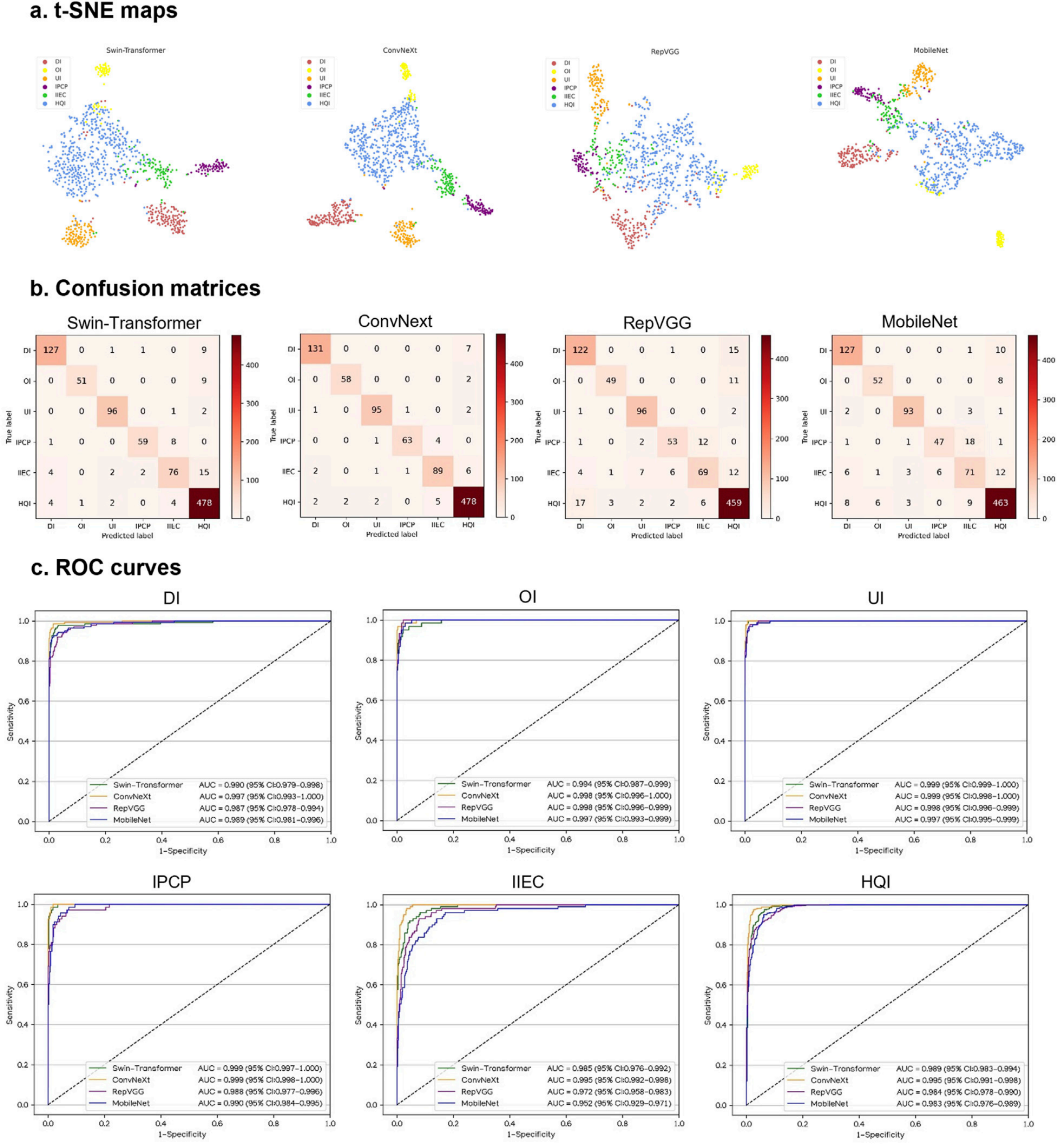
Performance of four different types of deep learning algorithms in a validation set. **(A)**, t-distributed stochastic neighbor embedding (t-SNE) maps. **(B)**, Confusion matrices. **(C)**, Receiver operating characteristic (ROC) curves; AUC, area under the curve; DI, defocused image; OI, overexposed image; UI, underexposed image; IPCP, image of poor cornea position; IIEC, image of incompletely exposed cornea; HQI, high-quality image.

This study utilized four DL algorithms (Swin-Transformer, ConvNeXt, RepVGG, and MobileNet) to train models designed for the discrimination of defocused images, overexposed images, underexposed images, PCP images, IEC images, and high-quality images. The t-SNE analysis revealed that ConvNeXt exhibited a superior capability to distinguish the embedding features of each class compared to Swin-Transformer, RepVGG, and MobileNet (Figure 3A).

In Figures 3B, C, the performance of the four algorithms on a validation set is depicted, highlighting ConvNeXt as the top-performing algorithm. The details, including sensitivities, specificities, and accuracies of the algorithms, are presented in Supplementary Table 2.

The best algorithm, ConvNeXt, attained an AUC of 0.997 (95% CI, 0.993–1.000), with a sensitivity of 94.9% (95% CI, 91.3–98.6) and a specificity of 99.5% (95% CI, 99.0–100) in the identification of defocused images. For discriminating overexposed images from the other categories, the ConvNeXt achieved an AUC of 0.998 (95% CI, 0.996–1.000), with a sensitivity of 96.7% (95% CI, 92.1–100) and a specificity of 99.8% (95% CI, 99.5–100). For distinguishing underexposed images from the other categories, the ConvNeXt achieved an AUC of 0.999 (95% CI, 0.998–1.000), with a sensitivity of 96.0% (95% CI, 92.1–99.8) and a specificity of 99.5% (95% CI, 99.1–100). For identifying PCP images, the ConvNeXt attained an AUC of 0.999 (95% CI, 0.998–1.000), with a sensitivity of 92.6% (95% CI, 86.4–98.9) and a specificity of 99.9% (95% CI, 99.7–100). For detecting ICE images, the ConvNeXt achieved an AUC of 0.995 (95% CI, 0.992–0.998), with a sensitivity of 89.9% (95% CI, 84.0–95.8) and a specificity of 98.9% (95% CI, 98.3–99.6). For distinguishing high-quality images from the other categories, the ConvNeXt attained an AUC of 0.995 (95% CI, 0.991–0.998), with a sensitivity of 97.8% (95% CI, 96.4–99.1) and a specificity of 96.8% (95% CI, 95.2–98.4). In comparison to the ground truth of the validation set, the unweighted Cohen’s kappa coefficient for ConvNeXt was 0.940 (95% CI, 0.922–0.958).

### Performance of DL models in an internal testing set

**FIGURE 4:**
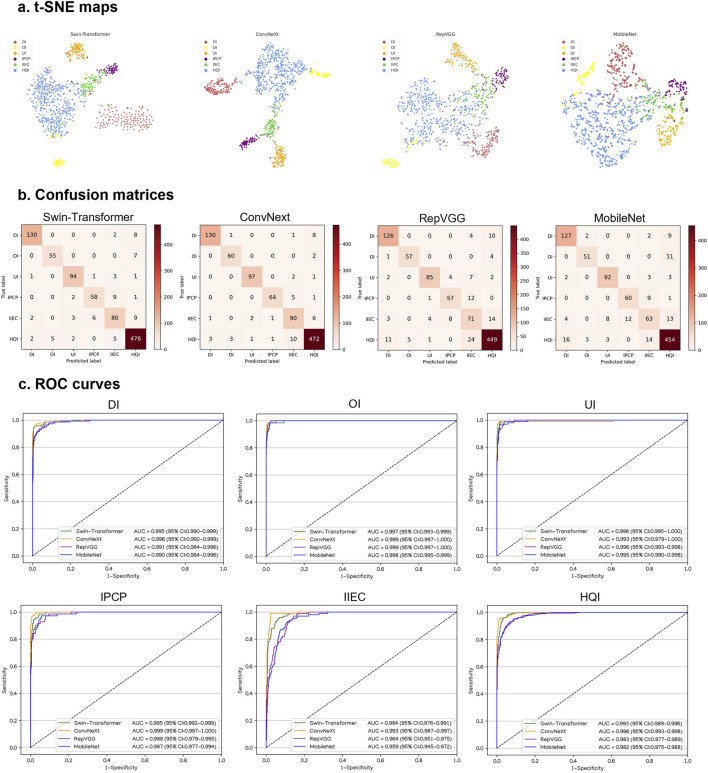
Performance of four different types of deep learning algorithms in an internal testing set. **(A)**, t-distributed stochastic neighbor embedding (t-SNE) maps. **(B)**, Confusion matrices. **(C)**, Receiver operating characteristic (ROC) curves; AUC, area under the curve; DI, defocused image; OI, overexposed image; UI, underexposed image; IPCP, image of poor cornea position; IIEC, image of incompletely exposed cornea; HQI, high-quality image.

In an internal testing set, the t-SNE technique also revealed that ConvNeXt exhibited more distinguishable features for each category compared to Swin-Transformer, RepVGG, and MobileNet (Figure 4A). Correspondingly, the confusion matrices (Figure 4B) and ROC curves (Figure 4C) of these four algorithms showed that ConvNeXt had the best performance. Additional details regarding the discriminative performance of the algorithms in the internal testing set are outlined in Supplementary Table 3.

For the classification of defocused images, overexposed images, underexposed images, PCP images, IEC images, and high-quality images, the optimal algorithm, ConvNeXt, attained AUCs of 0.996 (95% CI, 0.992–0.999), 0.999 (95% CI, 0.997–1.000), 0.993 (95% CI, 0.979–1.000), 0.999 (95% CI, 0.997–1.000), 0.993 (95% CI, 0.987–0.997), and 0.996 (95% CI, 0.993–0.998), respectively. The corresponding sensitivities were 92.9% (95% CI, 88.6–97.1), 96.8% (95% CI, 92.4–100), 97.0% (95% CI, 93.7–100), 91.4% (95% CI, 84.9–98.0), 90.0% (95% CI, 84.1–95.9), and 96.3% (95% CI, 94.7–98.0), and the corresponding specificities were 99.5% (95% CI, 99.0–100), 99.7% (95% CI, 99.3–100), 99.7% (95% CI, 99.3–100), 99.7% (95% CI, 99.3–100), 98.1% (95% CI, 97.2–99.0), and 96.4% (95% CI, 94.7–98.1). In comparison to the ground truth of the internal testing set, the unweighted Cohen’s kappa coefficient for ConvNeXt was 0.926 (95% CI, 0.906–0.946).

### Performance of DL models in external testing sets

**FIGURE 5:**
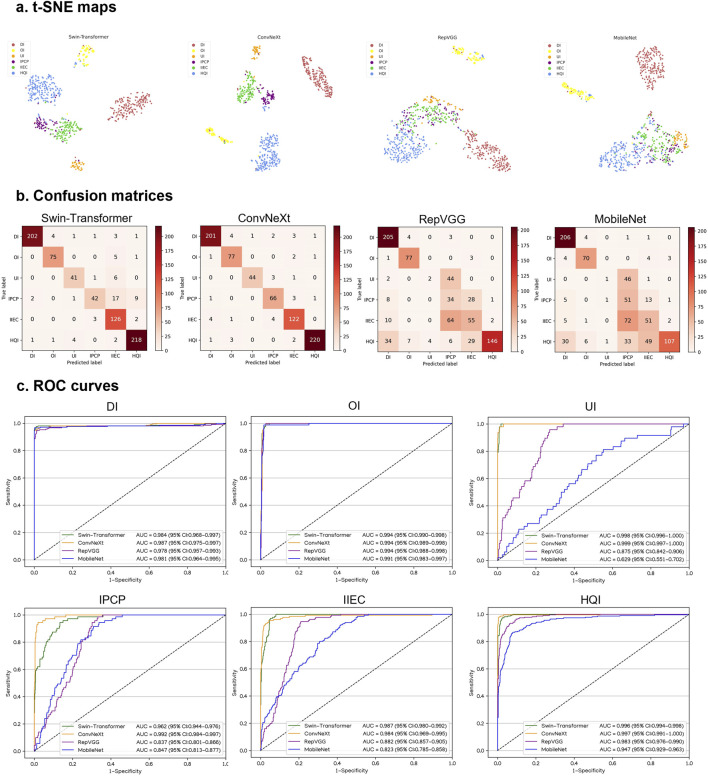
Performance of four different types of deep learning algorithms in a VIVO external testing set. **(A)**, t-distributed stochastic neighbor embedding (t-SNE) maps. **(B)**, Confusion matrices. **(C)**, Receiver operating characteristic (ROC) curves; AUC, area under the curve; DI, defocused image; OI, overexposed image; UI, underexposed image; IPCP, image of poor cornea position; IIEC, image of incompletely exposed cornea; HQI, high-quality image.

**FIGURE 6:**
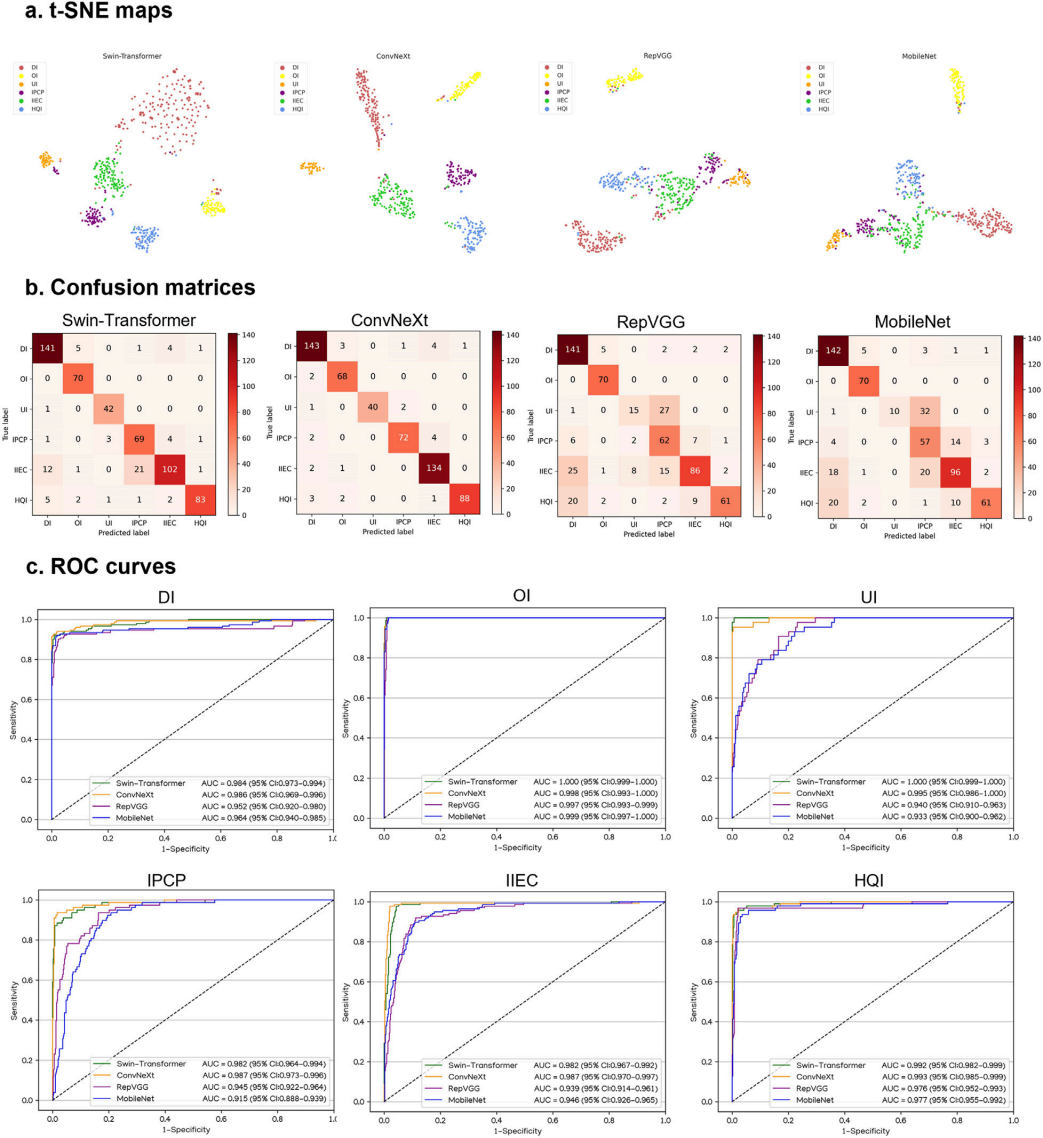
Performance of four different types of deep learning algorithms in a XIAOMI external testing set. **(A)**, t-distributed stochastic neighbor embedding (t-SNE) maps. **(B)**, Confusion matrices. **(C)**, Receiver operating characteristic (ROC) curves; AUC, area under the curve; DI, defocused image; OI, overexposed image; UI, underexposed image; IPCP, image of poor cornea position; IIEC, image of incompletely exposed cornea; HQI, high-quality image.

Similarly, *in VIVO* and XIAOMI external testing sets, the t-SNE maps, confusion matrices, and ROC curves denoted that ConvNeXt performs better than other algorithms (Figures 5, 6). The details regarding the performance of these four algorithms in the external testing sets are presented in Supplementary Tables 4, 5. In the VIVO external testing set, the best algorithm, ConvNeXt, attained AUCs of 0.987 (95% CI, 0.975–0.997), 0.994 (95% CI, 0.989–0.998), 0.999 (95% CI, 0.997–1.000), 0.992 (95% CI, 0.984–0.997), 0.984 (95% CI, 0.969–0.995), and 0.997 (95% CI, 0.991–1.000) for the identification of defocused images, overexposed images, underexposed images, PCP images, IEC images, and high-quality images, respectively. In the XIAOMI external testing sets, ConvNeXt achieved AUCs of 0.986 (95% CI, 0.969–0.996), 0.998 (95% CI, 0.993–1.000), 0.995 (95% CI, 0.986–1.000), 0.987 (95% CI, 0.973–0.996), 0.987 (95% CI, 0.970–0.997), and 0.993 (95% CI, 0.985–0.999) for the discrimination among defocused images, overexposed images, underexposed images, PCP images, IEC images, and high-quality images, respectively. In comparison to the ground truth of the VIVO and XIAOMI external testing sets, the unweighted Cohen’s kappa coefficients for ConvNeXt were 0.936 (95% CI, 0.916–0.955) and 0.937 (95% CI, 0.915–0.960), respectively.

### Visualization heatmap

**FIGURE 7:**
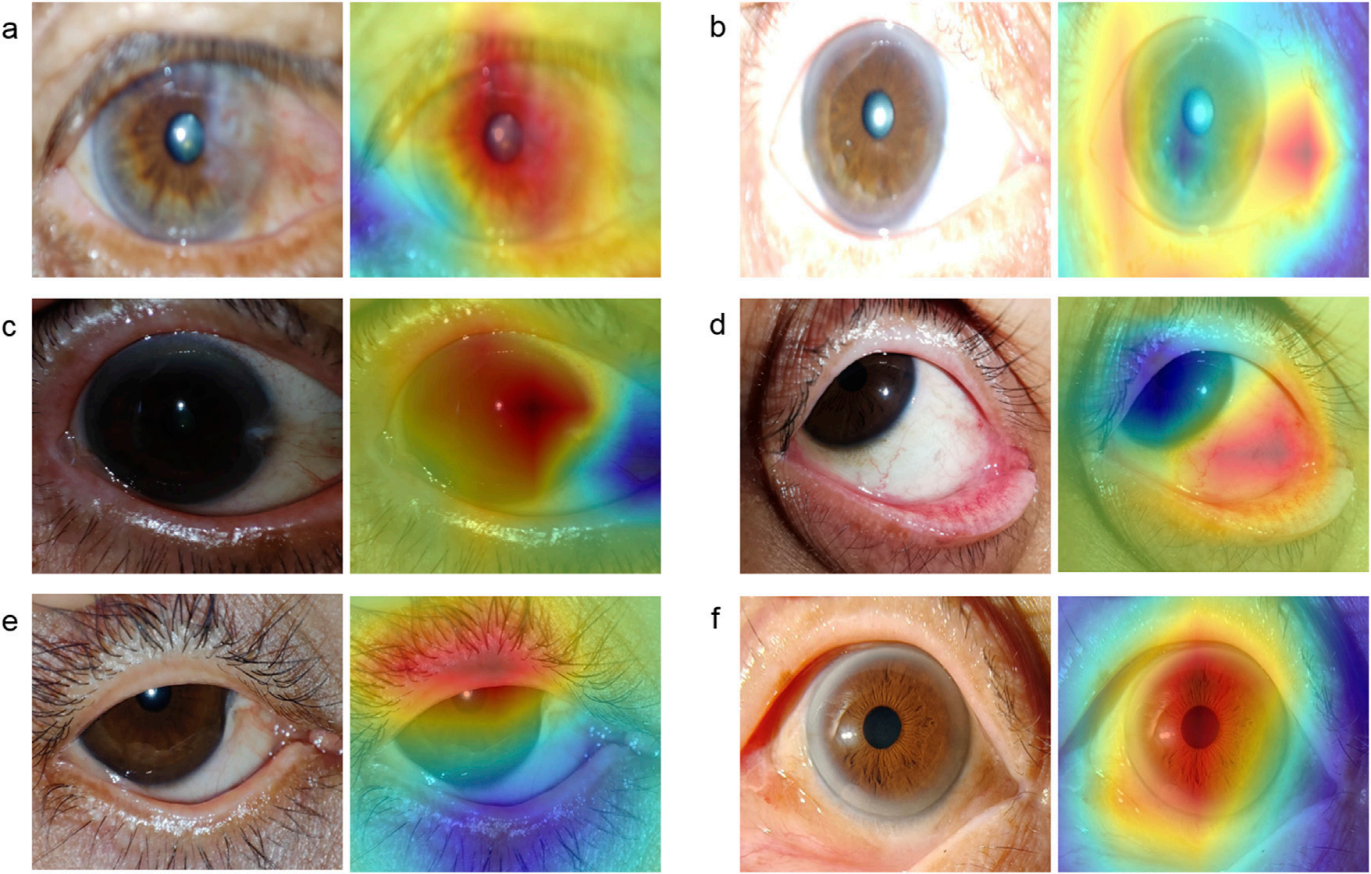
Typical heatmap examples of a defocused image, overexposed image, underexposed image, image of poor cornea position, image of incompletely exposed cornea, and high-quality image. The original image (left) and its corresponding heatmap (right) of each class are presented in pairs. **(A)**, defocused image. **(B)**, overexposed image. **(C)**, underexposed image. **(D)**, image of poor cornea position. **(E)**, image of incompletely exposed cornea. **(F)**, high-quality image.

To illustrate the image regions that play a significant role in influencing DeepMonitoring’s decision, we generated heatmaps by superimposing a visualization layer at the end of the convolutional neural networks. For defocused images, the heatmaps highlighted regions with blurriness. For overexposed images, the heatmaps displayed highlighted visualization on regions with excessive brightness. For underexposed images, the heatmaps displayed highlighted visualization on regions with insufficient brightness. For PCP images, the heatmaps highlighted the regions of the exposed conjunctiva. For IEC images, the heatmaps emphasized the regions of the eyelid that obscured the cornea. For high-quality images, the heatmaps showcased emphasized visualizations on the entire cornea. Examples of heatmaps for defocused images, overexposed images, underexposed images, PCP images, IEC images, and high-quality images are shown in Figure 7.

### Evaluation of the AI diagnostic system in low-quality and high-quality images

The AI diagnostic system attained AUCs of 0.849 (95% CI, 0.805–0.890), 0.806 (95% CI, 0.746–0.859), and 0.644 (95% CI, 0.581–0.701) for identifying keratitis, other corneal abnormalities, and normal cornea in low-quality images, respectively. In contrast, the system achieved corresponding AUCs of 0.973 (95% CI, 0.945–0.992), 0.979 (95% CI, 0.963–0.992), and 0.975 (95% CI, 0.957–0.991) in high-quality images. The ROC curves, confusion matrices, and t-SNE maps of the system in low-quality and high-quality images are presented in Supplementary Figure 2. The overall performance of the system in low-quality images was significantly worse than that in high-quality images (Supplementary Table 6). The accuracies of the AI diagnostic system in defocused images, overexposed images, underexposed images, PCP images, and IEC images were 56.7% (95% CI, 44.1–69.2), 68.3% (95% CI, 56.6–80.1), 60.0% (95% CI, 47.6–72.4), 48.3% (95% CI, 35.7–61.0), and 40.0% (95% CI, 27.6–52.4), respectively, indicating that the IEC images had the most significant negative impact on the system’s ability to detect keratitis, other corneal abnormalities, and normal cornea (average AUC = 0.642) (Supplementary Figure 3).

## Discussion

**FIGURE 8:**
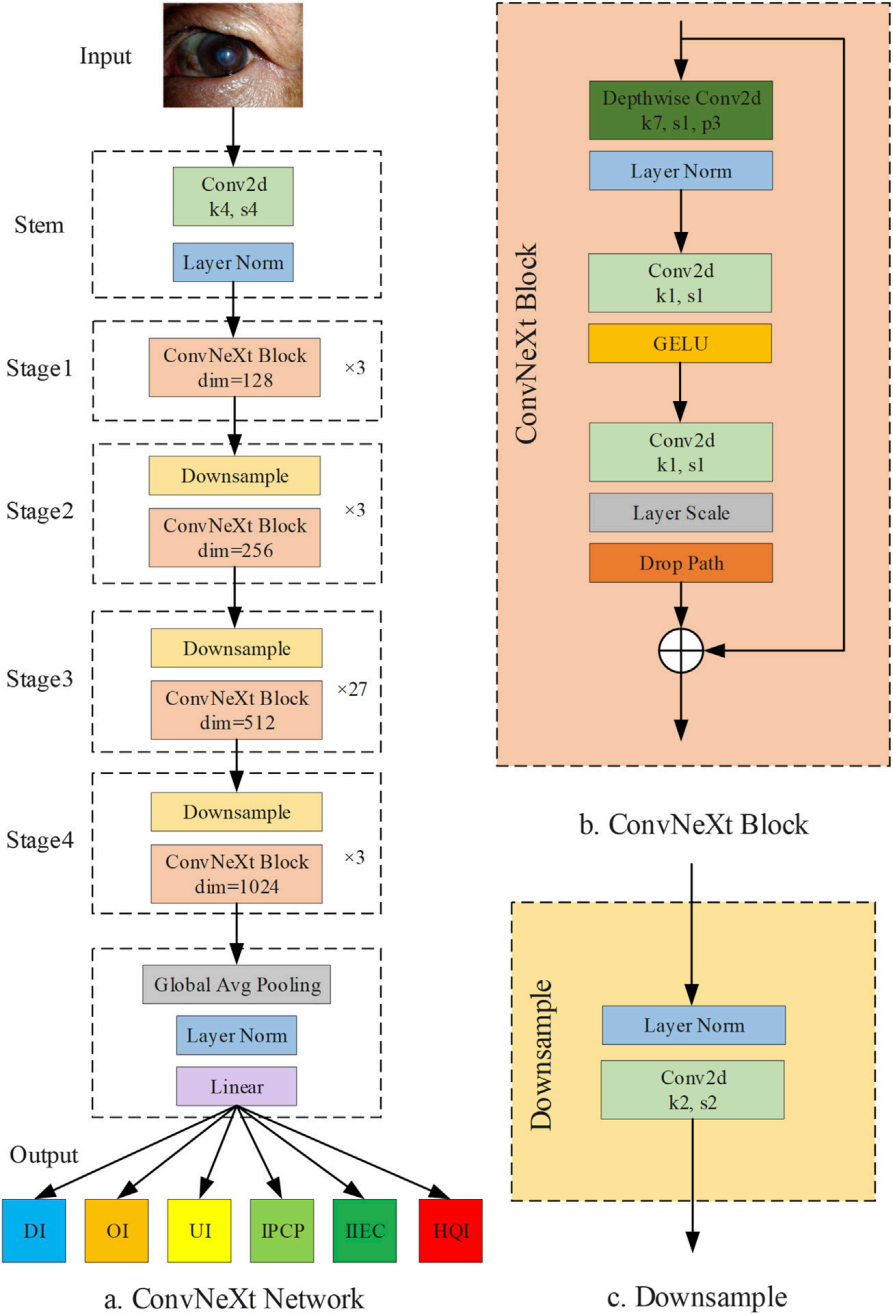
Diagram of DeepMonitoring. **(A)** Framwork of the ConvNeXt Network. **(B)** Structure of the ConvNeXt block. **(C)** Structure of the downsample layer; DI, defocused image; OI, overexposed image; UI, underexposed image; IPCP, image of poor cornea position; IIEC, image of incompletely exposed cornea; HQI, high-quality image.

A smartphone-based AI diagnostic system for self-detecting and monitoring corneal diseases in high-risk individuals is a valuable clinical tool that can help reduce the incidence of corneal blindness. This is because smartphones are now affordable and widely accessible. However, the generation of low-quality cornea images through smartphones is often unavoidable in real-world settings, particularly for non-professionals. This limitation decreases the accuracy of an AI diagnostic system, thereby hindering the widespread adoption and promotion of this system. Therefore, an image quality assessment process prior to the smartphone-based AI diagnostic system is indispensable. In this study, we established a DL system, DeepMonitoring (Figure 8), for detecting and filtering out low-quality cornea images produced by smartphones, and evaluated it on three image datasets derived from varying brands of smartphones. DeepMonitoring showed good performance across the validation, internal, and external testing sets (all AUCs over 0.98), demonstrating its robustness and broad generalizability. Additionally, the unweighted Cohen’s kappa coefficients revealed substantial agreement between the outputs of DeepMonitoring and the ground truth across all datasets (all over 0.92), further affirming the effectiveness of our DeepMonitoring.

Apart from detecting low-quality images, our DeepMonitoring can identify the causes (e.g., defocus, overexposure, and underexposure) of low-quality images with high accuracy. When a smartphone produces a low-quality image, our DeepMonitoring would provide targeted guidance to assist an operator in recapturing a high-quality image in a timely and effective manner. This process ensures that only high-quality images are transferred to a subsequent AI diagnostic system, enhancing the system’s performance in real-world settings. Besides, our DeepMonitoring can also be used to educate novice operators to improve their skills in obtaining high-quality cornea images via smartphones. These characteristics of DeepMonitoring would help the spread of smartphone-based AI diagnostic systems in clinical practice, empowering high-risk individuals to proactively identify their own corneal diseases at an early stage.

The interpretability of AI systems needs to be elucidated prior to they are applied in real-world clinical settings (Keel et al., 2019; Rudin, 2019). To elucidate the decision-making process of DeepMonitoring, heatmaps were created to highlight the crucial areas that the system employed to distinguish defocused images, overexposed images, underexposed images, PCP images, IEC images, and high-quality images. Our results indicated that the heatmaps showcased highlighted visualization on regions with blurriness in defocused images, on regions with excessive brightness in overexposed images, on regions with insufficient brightness in underexposed images, on regions of exposed conjunctiva in PCP images, on eyelid regions in IEC images, and on regions of the clear cornea in high-quality images. This interpretability feature of DeepMonitoring would enhance its applicability in real-world settings, as operators can comprehend how DeepMonitoring arrives at its final output.

The performance of our previously established AI diagnostic system in classifying keratitis, other corneal abnormalities, and normal cornea showed a significant decline in low-quality cornea images compared to high-quality images (Supplementary Table 6). This result proves the importance and indispensability of our DeepMonitoring for the smartphone-based AI diagnostic system. In addition, we found that the AI diagnostic system’s performance showed varying degrees of decline on defocused images, overexposed images, underexposed images, PCP images, and IEC images, among which, the most significant decline was observed on IEC images (Supplementary Figure 3). This result indicates that the patient’s eyes should be kept as wide open as possible while taking images of the cornea with a smartphone.

Our work has several crucial features. First of all, to the best of our knowledge, we constructed the first intelligent image quality monitoring system, DeepMonitoring, for automatically discerning and filtering out low-quality cornea images produced by smartphones. Second, our DeepMonitoring can identify the cause of a low-quality image and guide an operator to adopt a precise solution to obtain a high-quality image in a timely manner. In addition, the visual analysis of DeepMonitoring’s decision-making process was conducted using a Grad-CAM approach to gain the trust of human experts, which may promote the clinical implementation of this system. Fourth, to enhance the performance in distinguishing among different types of low-quality images, this study utilized substantial datasets for training and validating DeepMonitoring, consisting of 7,717 smartphone-based cornea images. Finally, our datasets were derived from two distinct clinical settings employing different smartphone brands and thus representing multiple scenarios in the real world.

Despite these impressive outcomes, our DeepMonitoring presents several limitations. First, DeepMonitoring is limited to classifying low-quality images and providing guidance for recapture, with no inherent capability to enhance the image quality. Further investigation is warranted to explore effective methods for improving the quality of cornea images captured by smartphones. In addition, as our DeepMonitoring was exclusively trained and tested on a Chinese population from various geographic regions, additional validation may be required to assess its applicability to other racial groups. In the near future, we expect to assess the performance of DeepMonitoring in various countries.

In conclusion, our DeepMonitoring showed reliable performance in discriminating among defocused images, overexposed images, underexposed images, PCP images, IEC images, and high-quality images. DeepMonitoring would promptly alert operators if an image’s quality is too low to rule out keratitis and other corneal abnormalities and assist them in retaking the image until it meets the quality requirements. We believe that DeepMonitoring holds great potential to facilitate the implementation of smartphone-based AI diagnostic systems in real-world settings, particularly for self-screening of corneal diseases.

## Data Availability

The original contributions presented in the study are included in the article/Supplementary Material, further inquiries can be directed to the corresponding authors.
