# Supplementary material for: DeepMonitoring: a deep learning-based monitoring system for assessing the quality of cornea images captured by smartphones
Source: Front Cell Dev Biol. 2024 Aug 27;12:1447067. doi: 10.3389/fcell.2024.1447067 (PMC11385315; doi:10.3389/fcell.2024.1447067)
Supplement: Supplementary file 1 [file Table1.docx]

**Title: DeepMonitoring: a deep learning-based monitoring system for assessing the quality of cornea images captured by smartphones**

**Supplementary Information**

**Supplementary Figure 1. Typical examples of keratitis images.**

**Supplementary Figure 2. Performance of the artificial intelligence diagnostic system for the detection of keratitis, other corneal abnormalities, and normal cornea in low-quality and high-quality images.** a, Receiver operating characteristic (ROC) curves. b, Confusion matrices. c, t-distributed stochastic neighbor embedding (t-SNE) maps. AUC, area under the curve. “Others” refers to other corneal abnormalities. “Normal” refers to normal cornea. HQ refers to high-quality images. LQ refers to low-quality images.

**Supplementary Figure 3. Performance of the artificial intelligence (AI) diagnostic system in different types of low-quality images.** a, Overall accuracies of the AI diagnostic system in different types of low-quality images. b, Receiver operating characteristic (ROC) curves of the AI diagnostic system for detecting keratitis in different types of low-quality images. c, ROC curves of the AI diagnostic system for detecting other corneal abnormalities in different types of low-quality images. d, ROC curves of the AI diagnostic system for detecting normal cornea in different types of low-quality images. AUC, area under the curve. DI, defocused image. OI, overexposed image. UI, underexposed image. IPCP, image of poor cornea position. IIEC, image of incompletely exposed cornea.

**Supplementary Table** **1. Characteristics of the trained deep learning models.**

**Supplementary Table 2. Detailed performance of deep learning algorithms in a validation set.**

**Supplementary Table 3. Detailed performance of deep learning algorithms in an internal testing set.**

**Supplementary Table 4. Detailed performance of deep learning algorithms in a VIVO external testing set.**

**Supplementary Table 5. Detailed performance of deep learning algorithms in a XIAOMI external testing set.**

**Supplementary Table 6. Detailed performance of the AI diagnostic system in low-quality and high-quality images for discerning keratitis, other corneal abnormalities, and normal cornea.**


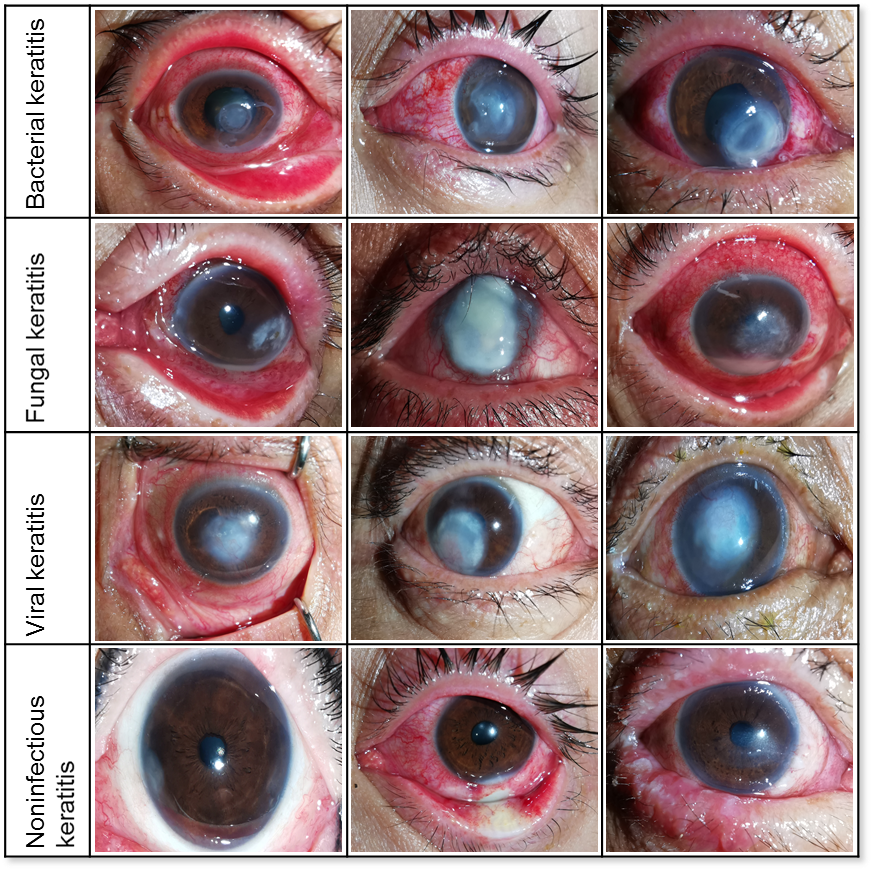


**Supplementary Figure 1.** **Typical examples of keratitis images.**


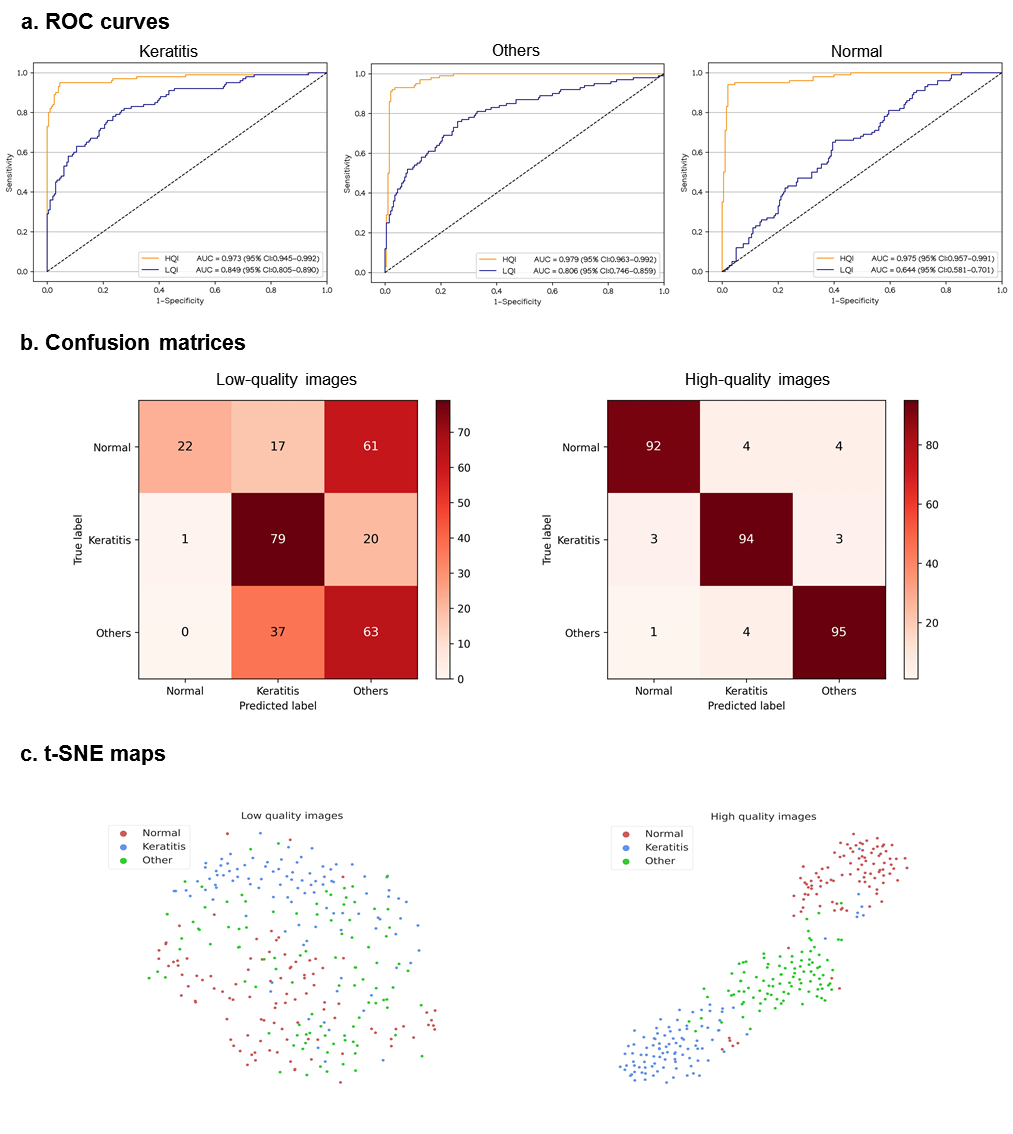


**Supplementary Figure 2. Performance of the** **artificial intelligence diagnostic system for the detection of keratitis, other corneal abnormalities, and normal cornea in low-quality and high-quality images.** a, Receiver operating characteristic (ROC) curves. b, Confusion matrices. c, t-distributed stochastic neighbor embedding (t-SNE) maps. AUC, area under the curve. “Others” refers to other corneal abnormalities. “Normal” refers to normal cornea. HQ refers to high-quality images. LQ refers to low-quality images.


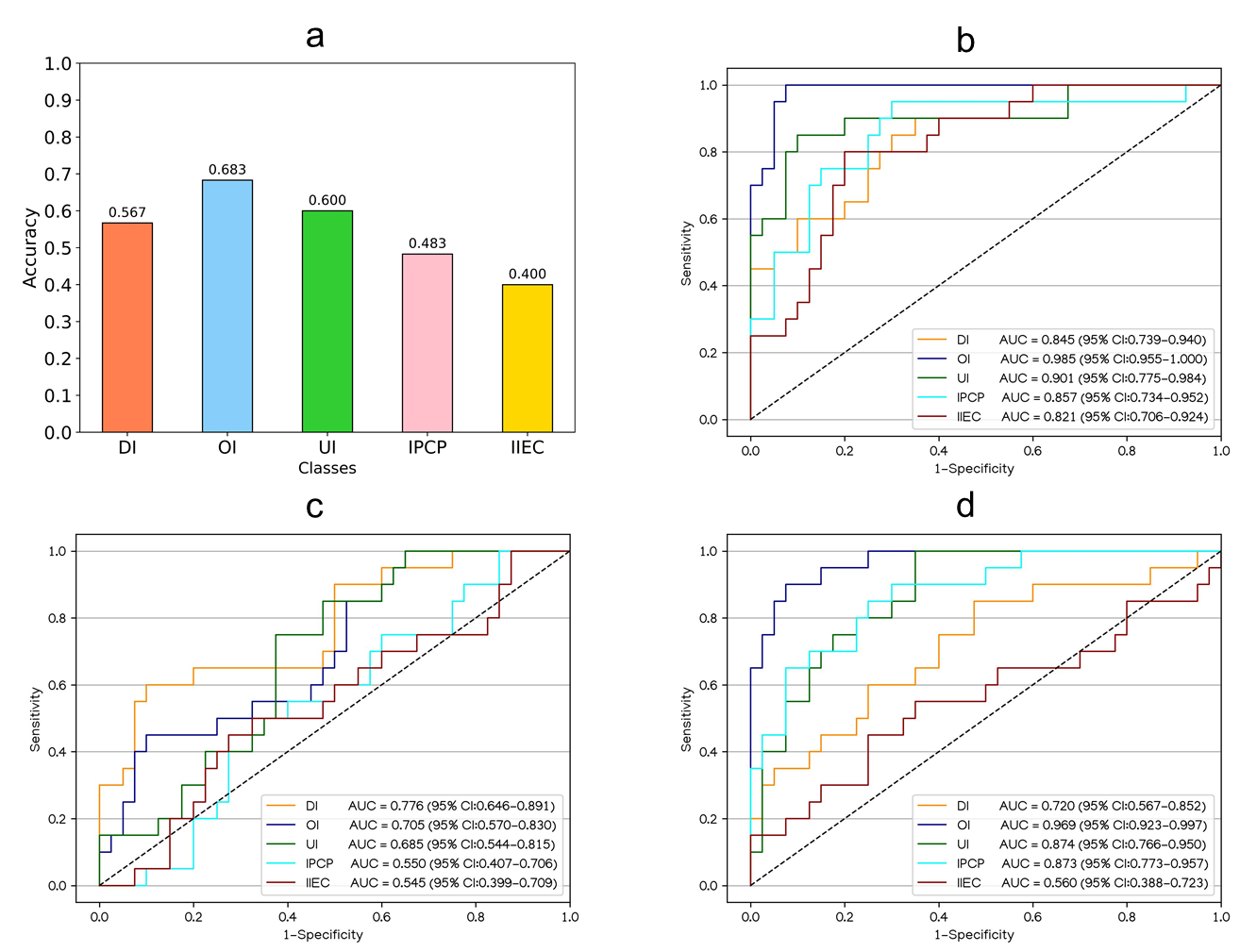


**Supplementary Figure 3. Performance of** **the artificial intelligence (AI) diagnostic system in different types of low-quality images.** a, Overall accuracies of the AI diagnostic system in different types of low-quality images. b, Receiver operating characteristic (ROC) curves of the AI diagnostic system for detecting keratitis in different types of low-quality images. c, ROC curves of the AI diagnostic system for detecting other corneal abnormalities in different types of low-quality images. d, ROC curves of the AI diagnostic system for detecting normal cornea in different types of low-quality images. AUC, area under the curve. DI, defocused image. OI, overexposed image. UI, underexposed image. IPCP, image of poor cornea position. IIEC, image of incompletely exposed cornea.

**Supplementary Table 1. Characteristics of the trained deep learning models.**

|  | Size | Trainable parameters | Training Time | Testing Time |
| --- | --- | --- | --- | --- |
| Swin-Transformer | 695.8MB | 8.8e+07 | 5.09h | 0.340s |
| ConvNeXt | 1051.2 MB | 8.9e+07 | 4.72h | 0.325s |
| RepVGG | 240.9 MB | 1.3e+07 | 4.26h | 0.298s |
| MobileNet | 32.9 MB | 4.0e+06 | 4.23h | 0.317s |

Training time indicates the running time of each model in the whole training process. Testing time indicates the average time that the model needs in testing every image. MB = Mbyte.

**Supplementary Table 2. Detailed performance of deep learning algorithms in a validation set.**

| One-vs.-rest classification | Accuracy (95% CI) | Sensitivity (95% CI) | Specificity (95% CI) |
| --- | --- | --- | --- |
| DI vs. OI + UI + IPCP + IIEC + HQI | | | |
| Swin-Transformer | 97.9% (97.0–98.8) | 92.0% (87.5–96.5) | 98.9% (98.2–99.6) |
| ConvNeXt | 98.8% (98.2–99.5) | 94.9% (91.3–98.6) | 99.5% (99.0–100) |
| RepVGG | 96.0% (94.8–97.3) | 88.4% (83.1–93.7) | 97.3% (96.2–98.4) |
| MobileNet | 97.3% (96.2–98.3) | 92.0% (87.5–99.5) | 98.2% (97.2–99.1) |
| OI vs. DI + UI + IPCP + IIEC + HQI | | | |
| Swin-Transformer | 98.8% (98.2–99.5) | 85.0% (76.0–94.0) | 99.8% (99.5–100) |
| ConvNeXt | 99.6% (99.2–100) | 96.7% (92.1–100) | 99.8% (99.5–100) |
| RepVGG | 98.4% (97.6–99.2) | 81.7% (71.9–91.5) | 99.6% (99.1–100) |
| MobileNet | 98.4% (97.6–99.2) | 86.7% (78.1–95.3) | 99.2% (98.6–99.8) |
| UI vs. DI + OI + IPCP + IIEC + HQI | | | |
| Swin-Transformer | 99.2% (98.6–99.7) | 97.0% (93.6–100) | 99.4% (98.9–99.9) |
| ConvNeXt | 99.2% (98.6–99.7) | 96.0% (92.1–99.8) | 99.5% (99.1–100) |
| RepVGG | 98.4% (97.6–99.2) | 97.0% (93.6–100) | 98.6% (97.8–99.4) |
| MobileNet | 98.6% (97.9–99.4) | 93.9% (89.2–98.6) | 99.2% (98.6–99.8) |
| IPCP vs. DI + OI + UI + IIEC + HQI | | | |
| Swin-Transformer | 98.8% (98.2–99.5) | 86.8% (78.7–94.8) | 99.8% (99.5–100) |
| ConvNeXt | 99.4% (98.9–99.9) | 92.6% (86.4–98.9) | 99.9% (99.7–100) |
| RepVGG | 97.6% (96.6–98.6) | 77.9% (68.1–87.8) | 99.1% (98.5–99.7) |
| MobileNet | 97.2% (96.1–98.2) | 69.1% (58.1–80.1) | 99.3% (98.8–99.9) |
| IIEC vs. DI + OI + UI + IPCP + HQI | | | |
| Swin-Transformer | 96.3% (95.1–97.5) | 76.8% (68.4–85.1) | 98.6% (97.8–99.4) |
| ConvNeXt | 98.0% (97.1–98.9) | 89.9% (84.0–95.8) | 98.9% (98.3–99.6) |
| RepVGG | 95.0% (93.6–96.4) | 69.7% (60.6–78.7) | 97.9% (96.9–98.9) |
| MobileNet | 94.1% (92.6–95.6) | 71.7% (62.8–80.6) | 96.7% (95.5–97.9) |
| HQI vs. DI + OI + UI + IPCP + IIEC | | | |
| Swin-Transformer | 95.4% (94.1–96.7) | 97.8% (96.4–99.1) | 92.9% (90.5–95.2) |
| ConvNeXt | 97.3% (96.2–98.3) | 97.8% (96.4–99.1) | 96.8% (95.2–98.4) |
| RepVGG | 92.9% (91.2–94.5) | 93.9% (91.7–96.0) | 91.8% (89.3–94.3) |
| MobileNet | 94.0% (92.5–95.5) | 94.7% (92.7–96.7) | 93.3% (91.0–95.6) |

CI, confidence interval. DI, defocused image. OI, overexposed image. UI, underexposed image. IPCP, image of poor cornea position. IIEC, image of incompletely exposed cornea. HQI, high-quality image.

**Supplementary Table 3. Detailed performance of deep learning algorithms in an internal testing set.**

| One-vs.-rest classification | Accuracy (95% CI) | Sensitivity (95% CI) | Specificity (95% CI) |
| --- | --- | --- | --- |
| DI vs. OI + UI + IPCP + IIEC + HQI | | | |
| Swin-Transformer | 98.5% (97.8–99.3) | 92.9% (88.6–97.1) | 99.5% (99.0–100) |
| ConvNeXt | 98.5% (97.8–99.3) | 92.9% (88.6–97.1) | 99.5% (99.0–100) |
| RepVGG | 96.6% (95.4–97.7) | 90.7% (85.9–95.5) | 97.6% (96.5–98.6) |
| MobileNet | 97.0% (95.9–98.1) | 90.0% (85.0–95.0) | 98.2% (97.3–99.1) |
| OI vs. DI + UI + IPCP + IIEC + HQI | | | |
| Swin-Transformer | 98.8% (98.1–99.5) | 88.7% (80.8–96.6) | 99.4% (99.0–99.9) |
| ConvNeXt | 99.5% (99.0–99.9) | 96.8% (92.4–100) | 99.7% (99.3–100) |
| RepVGG | 98.5% (97.8–99.3) | 82.3% (72.7–91.8) | 99.7% (99.3–100) |
| MobileNet | 99.0% (98.3–99.6) | 91.9% (85.2–98.7) | 99.4% (99.0–99.9) |
| UI vs. DI + OI + IPCP + IIEC + HQI | | | |
| Swin-Transformer | 98.6% (97.9–99.4) | 94.0% (89.3–98.7) | 99.2% (98.6–99.8) |
| ConvNeXt | 99.4% (98.9–99.9) | 97.0% (93.7–100) | 99.7% (99.3–100) |
| RepVGG | 98.0% (97.1–98.9) | 92.0% (86.7–97.3) | 98.7% (98.0–99.5) |
| MobileNet | 97.8% (96.9–98.7) | 85.0% (78.0–92.0) | 99.3% (98.7–99.9) |
| IPCP vs. DI + OI + UI + IIEC + HQI | | | |
| Swin-Transformer | 98.1% (97.3–99.0) | 82.9% (74.0–91.7) | 99.3% (98.8–99.9) |
| ConvNeXt | 99.1% (98.5–99.7) | 91.4% (84.9–98.0) | 99.7% (99.3–100) |
| RepVGG | 97.5% (96.5–98.5) | 85.7% (77.5–93.9) | 98.4% (97.6–99.2) |
| MobileNet | 97.8% (96.9–98.7) | 81.4% (72.3–90.5) | 99.1% (98.5–99.7) |
| IIEC vs. DI + OI + UI + IPCP + HQI | | | |
| Swin-Transformer | 96.3% (95.1–97.5) | 80.0% (72.2–87.8) | 98.1% (97.2–99.0) |
| ConvNeXt | 97.3% (96.3–98.3) | 90.0% (84.1–95.9) | 98.1% (97.2–99.0) |
| RepVGG | 93.6% (92.0–95.1) | 63.0% (53.5–72.5) | 97.1% (96.0–98.2) |
| MobileNet | 92.8% (91.2–94.5) | 71.0% (62.1–79.9) | 95.4% (94.0–96.8) |
| HQI vs. DI + OI + UI + IPCP + IIEC | | | |
| Swin-Transformer | 95.9% (94.7–97.2) | 97.1% (95.7–98.6) | 94.7% (92.7–96.7) |
| ConvNeXt | 96.4% (95.2–97.5) | 96.3% (94.7–98.0) | 96.4% (94.7–98.1) |
| RepVGG | 92.7% (91.1–94.4) | 92.7% (90.3–95.0) | 92.8% (90.5–95.1) |
| MobileNet | 92.8% (91.2–94.5) | 91.6% (89.2–94.1) | 94.1% (91.9–96.2) |

CI, confidence interval. DI, defocused image. OI, overexposed image. UI, underexposed image. IPCP, image of poor cornea position. IIEC, image of incompletely exposed cornea. HQI, high-quality image.

**Supplementary Table 4. Detailed performance of deep learning algorithms in a VIVO external testing set.**

| One-vs.-rest classification | Accuracy (95% CI) | Sensitivity (95% CI) | Specificity (95% CI) |
| --- | --- | --- | --- |
| DI vs. OI + UI + IPCP + IIEC + HQI | | | |
| Swin-Transformer | 98.3% (97.4–99.2) | 95.3% (92.4–98.1) | 99.5% (98.9–100) |
| ConvNeXt | 97.7% (96.6–98.7) | 94.8% (91.8–97.8) | 98.7% (97.8–99.7) |
| RepVGG | 91.9% (90.0–93.9) | 96.7% (94.3–99.1) | 90.1% (87.6–92.6) |
| MobileNet | 93.5% (91.8–95.2) | 97.2% (94.9–99.4) | 92.1% (89.9–94.3) |
| OI vs. DI + UI + IPCP + IIEC + HQI | | | |
| Swin-Transformer | 98.6% (97.7–99.4) | 92.6% (86.9–98.3) | 99.3% (98.6–99.9) |
| ConvNeXt | 98.4% (97.6–99.3) | 95.1% (90.3–99.8) | 98.8% (98.0–99.6) |
| RepVGG | 98.0% (97.1–99.0) | 95.1% (90.3–99.8) | 98.4% (97.5–99.3) |
| MobileNet | 97.1% (96.0–98.3) | 86.4% (79.0–93.9) | 98.4% (97.5–99.3) |
| UI vs. DI + OI + IPCP + IIEC + HQI | | | |
| Swin-Transformer | 98.3% (97.4–99.2) | 85.4% (75.4–95.4) | 99.2% (98.5–99.8) |
| ConvNeXt | 99.3% (98.8–99.9) | 91.7% (83.8–99.5) | 99.9% (99.6–100) |
| RepVGG | 93.5% (91.8–95.2) | 4.2% (0.0–9.8) | 99.4% (98.9–100) |
| MobileNet | 93.6% (91.9–95.4) | 2.1% (0.0–6.1) | 99.7% (99.3–100) |
| IPCP vs. DI + OI + UI + IIEC + HQI | | | |
| Swin-Transformer | 95.6% (94.1–97.0) | 59.2% (47.7–70.6) | 99.3% (98.7–99.9) |
| ConvNeXt | 98.2% (97.2–99.1) | 93.0% (87.0–98.9) | 98.7% (97.9–99.5) |
| RepVGG | 80.0% (77.1–82.8) | 47.9% (36.3–59.5) | 83.2% (80.5–86.0) |
| MobileNet | 77.6% (74.7–80.6) | 71.8% (61.4–82.3) | 78.2% (75.2–81.3) |
| IIEC vs. DI + OI + UI + IPCP + HQI | | | |
| Swin-Transformer | 95.1% (93.5–96.6) | 96.2% (92.9–99.5) | 94.8% (93.1–96.5) |
| ConvNeXt | 97.4% (96.3–98.5) | 93.1% (88.8–97.5) | 98.3% (97.3–99.3) |
| RepVGG | 82.3% (79.6–85.0) | 42.0% (33.5–50.4) | 90.6% (88.3–92.9) |
| MobileNet | 80.8% (78.0–83.5) | 38.9% (30.6–47.3) | 89.3% (86.9–91.7) |
| HQI vs. DI + OI + UI + IPCP + IIEC | | | |
| Swin-Transformer | 97.3% (96.1–98.4) | 96.5% (94.1–98.9) | 97.6% (96.3–98.9) |
| ConvNeXt | 98.8% (98.1–99.6) | 97.3% (95.2–99.4) | 99.4% (98.8–100) |
| RepVGG | 89.2% (87.0–91.4) | 64.6% (58.4–70.8) | 99.4% (98.8–100) |
| MobileNet | 83.7% (81.1–86.4) | 47.3% (40.8–53.9) | 98.9% (98.0–99.8) |

CI, confidence interval. DI, defocused image. OI, overexposed image. UI, underexposed image. IPCP, image of poor cornea position. IIEC, image of incompletely exposed cornea. HQI, high-quality image.

**Supplementary Table 5. Detailed performance of deep learning algorithms in a XIAOMI external testing set.**

| One-vs.-rest classification | Accuracy (95% CI) | Sensitivity (95% CI) | Specificity (95% CI) |
| --- | --- | --- | --- |
| DI vs. OI + UI + IPCP + IIEC + HQI | | | |
| Swin-Transformer | 94.8% (93.0–96.6) | 92.8% (88.6–96.9) | 95.5% (93.5–97.5) |
| ConvNeXt | 96.7% (95.2–98.2) | 94.1% (90.3–97.8) | 97.6% (96.2–99.1) |
| RepVGG | 89.0% (86.5–91.6) | 92.8% (88.6–96.9) | 87.7% (84.5–90.8) |
| MobileNet | 90.8% (88.4–93.1) | 93.4% (89.5–97.4) | 89.8% (96.9–92.7) |
| OI vs. DI + UI + IPCP + IIEC + HQI | | | |
| Swin-Transformer | 98.6% (97.6–99.6) | 100% (100–100) | 98.4% (97.3–99.5) |
| ConvNeXt | 98.6% (97.6–99.6) | 97.1% (93.2–100) | 98.8% (97.9–99.8) |
| RepVGG | 98.6% (97.6–99.6) | 100% (100–100) | 98.4% (97.3–99.5) |
| MobileNet | 98.6% (97.6–99.6) | 100% (100–100) | 98.4% (97.3–99.5) |
| UI vs. DI + OI + IPCP + IIEC + HQI | | | |
| Swin-Transformer | 99.1% (98.4–99.9) | 97.7% (93.2–100) | 99.2% (98.5–100) |
| ConvNeXt | 99.5% (98.9–100) | 93.0% (85.4–100) | 100% (100–100) |
| RepVGG | 93.4% (91.3–95.4) | 34.9% (20.6–49.1) | 98.1% (97.0–99.3) |
| MobileNet | 94.3% (92.3–96.2) | 23.3% (10.6–35.9) | 100% (100–100)) |
| IPCP vs. DI + OI + UI + IIEC + HQI | | | |
| Swin-Transformer | 94.4% (92.5–96.3) | 88.5% (81.4–95.6) | 95.4% (93.5–97.2) |
| ConvNeXt | 98.4% (97.4–99.4) | 92.3% (86.4–98.2) | 99.4% (98.7–100) |
| RepVGG | 89.2% (86.7–91.7) | 79.5% (70.5–88.4) | 90.7% (88.2–93.3) |
| MobileNet | 86.6% (83.8–89.4) | 73.1% (63.2–82.9) | 88.7% (85.9–91.5) |
| IIEC vs. DI + OI + UI + IPCP + HQI | | | |
| Swin-Transformer | 92.2% (90.0–94.4) | 74.5% (67.1–81.8) | 97.7% (96.3–99.1) |
| ConvNeXt | 97.9% (96.7–99.1) | 97.8% (95.4–100) | 97.9% (96.6–99.3) |
| RepVGG | 88.0% (85.3–90.6) | 62.8% (54.7–70.9) | 95.9% (94.0–97.7) |
| MobileNet | 88.5% (85.9–91.1) | 70.1% (62.4–77.7) | 94.3% (92.1–96.5) |
| HQI vs. DI + OI + UI + IPCP + IIEC | | | |
| Swin-Transformer | 97.6% (96.3–98.8) | 88.3% (81.8–94.8) | 99.4% (98.7–100) |
| ConvNeXt | 98.8% (97.9–99.7) | 93.6% (88.7–98.6) | 99.8% (99.4–100) |
| RepVGG | 93.4% (91.3–95.4) | 64.9% (55.2–74.5) | 99.0% (98.1–99.9) |
| MobileNet | 93.2% (91.1–95.3) | 64.9% (55.2–74.5) | 98.8% (97.8–99.7) |

CI, confidence interval. DI, defocused image. OI, overexposed image. UI, underexposed image. IPCP, image of poor cornea position. IIEC, image of incompletely exposed cornea. HQI, high-quality image.

**Supplementary Table 6. Detailed performance of the AI diagnostic system in low-quality and high-quality images for discerning keratitis, other corneal abnormalities, and normal cornea.**

| One-vs.-Rest Classification | Low-quality images | High-quality images | *P* |
| --- | --- | --- | --- |
| **Keratitis vs. others + normal** | | | |
| Sensitivity (95% CI) | 79.0% (71.0-87.0) | 94.0% (89.3-98.7) | 0.004 |
| Specificity (95% CI) | 73.0% (66.8-79.2) | 96.0% (93.3-98.7) | <0.001 |
| Accuracy (95% CI) | 75.0% (70.1-79.9) | 95.3% (92.9-97.7) | <0.001 |
| **Others vs. keratitis + normal** | | | |
| Sensitivity (95% CI) | 63.0% (53.5-72.5) | 95.0% (90.7-99.3) | <0.001 |
| Specificity (95% CI) | 59.5% (52.7-66.3) | 96.5% (94.0-99.0) | <0.001 |
| Accuracy (95% CI) | 60.7% (55.1-66.2) | 96.0% (93.8-98.2) | <0.001 |
| **Normal vs. keratitis + others** | | | |
| Sensitivity (95% CI) | 22.0% (13.9-30.1) | 92.0% (86.7-97.3) | <0.001 |
| Specificity (95% CI) | 99.5% (98.5-100) | 98.0% (96.1-99.9) | 0.368 |
| Accuracy (95% CI) | 73.7% (68.7-78.7) | 96.0% (93.8-98.2) | <0.001 |

“Others” refers to other corneal abnormalities. “Normal” refers to normal cornea. CI, confidence interval. *P* denotes the p-value calculated between the low-quality images and high-quality images using the McNemar test.
